# MicroRNAs as Diagnostic and Prognostic Biomarkers in Ischemic Stroke—A Comprehensive Review and Bioinformatic Analysis

**DOI:** 10.3390/cells7120249

**Published:** 2018-12-06

**Authors:** Ceren Eyileten, Zofia Wicik, Salvatore De Rosa, Dagmara Mirowska-Guzel, Aleksandra Soplinska, Ciro Indolfi, Iwona Jastrzebska-Kurkowska, Anna Czlonkowska, Marek Postula

**Affiliations:** 1 Department of Experimental and Clinical Pharmacology, Medical University of Warsaw, Center for Preclinical Research and Technology CEPT, 02-097 Warsaw, Poland; ceren.eyileten-postula@wum.edu.pl (C.E.); dmirowska@wum.edu.pl (D.M.-G.); ola@soplinska.pl (A.S.); 2 Rheumatology Division, Hospital das Clinicas HCFMUSP, Universidade de Sao Paulo, Sao Paulo, SP 01246-903, Brazil; zofiawicik@gmail.com; 3 Division of Cardiology, Department of Medical and Surgical Sciences, “Magna Graecia” University, 88100 Catanzaro, Italy; saderosa@unicz.it (S.D.R.); indolfi@unicz.it (C.I.); 4 URT-CNR, Department of Medicine, Consiglio Nazionale delle Ricerche of IFC, Viale Europa S/N, 88100 Catanzaro, Italy; 5 2nd Department of Neurology, Institute of Psychiatry and Neurology, 02-957 Warsaw, Poland; ikurkowska@ipin.edu.pl (I.J.-K.); czlonkow@ipin.edu.pl (A.C.)

**Keywords:** miRNA, bioinformatic analysis, ischemic stroke, miRNA-gene target interaction, network, biomarker, diagnosis, prognosis

## Abstract

Stroke is the second-most common cause of death worldwide. The pathophysiology of ischemic stroke (IS) is related to inflammation, atherosclerosis, blood coagulation, and platelet activation. MicroRNAs (miRNAs) play important roles in physiological and pathological processes of neurodegenerative diseases and progression of certain neurological diseases, such as IS. Several different miRNAs, and their target genes, are recognized to be involved in the pathophysiology of IS. The capacity of miRNAs to simultaneously regulate several target genes underlies their unique value as diagnostic and prognostic markers in IS. In this review, we focus on the role of miRNAs as diagnostic and prognostic biomarkers in IS. We discuss the most common and reliable detection methods available and promising tests currently under development. We also present original results from bioinformatic analyses of published results, identifying the ten most significant genes (HMGB1, YWHAZ, PIK3R1, STAT3, MAPK1, CBX5, CAPZB, THBS1, TNFRSF10B, RCOR1) associated with inflammation, blood coagulation, and platelet activation and targeted by miRNAs in IS. Additionally, we created miRNA-gene target interaction networks based on Gene Ontology (GO) information derived from publicly available databases. Among our most interesting findings, miR-19a-3p is the most widely modulated miRNA across all selected ontologies and might be proposed as novel biomarker in IS to be tested in future studies.

## 1. Introduction

Stroke is the second-most common cause of death worldwide, accounting for almost 6.5 million stroke deaths each year. Approximately 795,000 strokes occur in the United States each year. On average, every 40 s, someone in the United States has a stroke, and on average, every 4 min, someone dies of a stroke [1,2]. In Europe, strokes account for 405,000 deaths (9%) in men and 583,000 (13%) deaths in women each year [3]. While hemorrhagic strokes accounts for 15% and a further 5% are due to unknown etiology, approximately 80% of all acute strokes are ischemic, mainly from large vessel occlusion due to either artery-to-artery embolism or cardiac embolism. [4,5]. Ischemic stroke (IS) is characterized by the sudden loss of blood circulation to an area of the brain, resulting in irreversible brain injury and subsequent neurologic deficits occurring already few minutes after the onset of ischemia [6].

MicroRNAs (miRNA) are small, endogenous, single-stranded, noncoding RNA molecules ranging in length from 18–25 nt that are found in eukaryotic cells. They regulate approximately 60% of the mammalian protein coding genes, primarily through the interaction with mRNAs. This effect is exerted by binding to complementary regions of messenger transcripts to repress their translation or less frequently inducing their degradation. Up to now, more than 5000 human miRNAs have been identified, and about 1000 are estimated to exist [7]. Several studies showed that miRNAs can be useful for the treatment of particular diseases. One advantage of miRNA-based therapies is that synthetic mimics and inhibitors can be used to alter endogenous miRNA levels in clinic. This approach has been utilized for cancer and hepatitis C virus therapy [8,9]. Apart from their treatment value, it was shown that expression of miRNAs plays important roles in many physiological and pathological processes, including epigenetics and neurodegenerative diseases and progression of specific neurological diseases [10,11]. It has been shown that some pathological processes of the central nervous system, and that Alzheimer’s disease, multiple sclerosis, and stroke are associated to specific alteration of circulating miRNAs [12]. This suggests that circulating miRNAs could be used as clinical biomarkers, to provide a sort of “liquid biopsy” taken from peripheral blood but providing information on pathophysiological processes going on in the brain and underlying stroke [13,14,15].

Potential advantages of this approach include the early diagnosis of acute cerebrovascular events, as already proven before with coronary artery disease, peripheral arterial disease and ischemia, acute myocardial infarction, myocarditis, heart failure, takotsubo cardiomyopathy in-stent restenosis, diabetes, or platelet dysfunction [7,16,17,18,19,20,21,22,23,24,25,26,27,28,29].

Several conventional methods were used by researchers in order to detect miRNAs such as, northern blotting, quantitative polymerase chain reaction (qPCR), and microarrays. However, each of these methods has its own individual limitations. Northern analysis was a widely used method for miRNA analyses, as it is easy to perform, specificity is relatively high, sequences with even partial homology can be used as hybridization probes, and the cost of running many gels is low once the equipment is set up [30]. However, northern blot technology also has disadvantages. For example, contamination due to radiolabeling, low detection efficiency, and poor sensitivity for oligonucleotide probes may complicate the measurements [31]. RT-PCR and microarrays allow reliable and effective detection for miRNAs. Techniques like northern blot and RT-PCR allow testing for only a few miRNAs per experiment. On the contrary, using miRNA microarray, it is possible to identify more than several hundred differentially regulated miRNAs in a particular disease or condition in one single run [7]. Several new technical developments have been brought about in the field of RT-PCR in order to further improve their efficiency. Among the others, the coupling with microfluidic cards should be mentioned, together with the digital droplet PCR, which allows very high analytical standards, minimizing reaction volumes [32]. Additionally, next-generation-sequencing technology has quickly emerged as the preferred platform for studying and discovering novel circulating miRNAs. It is possible to sequence multiple samples in one time by pooling with next-generation-sequencing and also possible to construct comprehensive expression profiles for every assessed sample, on the other hand potentially large investment in bioinformatics analysis, time and personnel are required [33,34]. Beside these techniques, there are new promising methodologies have been developed to detect miRNAs, for example, sensitive miRNA biosensors such as silicon nanowires, gold nanoparticles, silver nanoclusters, or conducting polymer/carbon nanotube hybrids [35,36,37,38].

Since circulating miRNAs can be detected by several techniques in plasma, serum, or whole blood, they have become the focus of interest as potential new diagnostic and prognostic biomarkers and tools for understanding their role in IS. Therefore, we systematically reviewed all available studies assessing the clinical usefulness of circulating miRNAs as diagnostic or prognostic markers in IS. Thus, in this article, we present an overview of the current knowledge on diagnostic and prognostic value of miRNAs related to IS based on human studies and report the results of a quantitative bioinformatic analysis highlighting the most promising miRNAs for clinical application in IS.

## 2. Circulating miRNAs and Stroke

Acute myocardial ischemia and IS share several common pathophysiological aspects. Previous studies implied that the circulating miRNAs could be exploited as diagnostic and/or prognostic indicators in several cardiovascular diseases (CVD) [27]. However, limited number of researches on role of miRNAs in IS and its relation to clinical outcomes were published.

### 2.1. PCR-Based Analysis for miRNA Expression

Quantitative reverse transcription PCR (RT-qPCR) technology is the gold standard for gene expression measurement. Several companies have developed qPCR-based assays for the detection of miRNA expression [39]. RT-qPCR technique allowed us to achieve high sensitivity, high specificity, and quantitative data of miRNA expression analysis. However, RT-qPCR is low-throughput profiling for a genome-wide miRNA-profiling assay [40].

In one of the first study, alterations in plasma miRNA levels in patients with IS were compared to healthy controls. The circulating levels of miR-30a, miR-126, and let-7b were analyzed in plasma samples of patients with IS obtained at different time-points both in acute phase (24 h from the onset of symptoms) and recovery phase (at 1 week, 4 weeks, 24 weeks, and 48 weeks after the episode). Circulating miR-30a and miR-126 expression were down regulated in IS patients regardless of etiology in all time points, except the last measurement at 48 weeks when it increase to baseline value. Interestingly, the expression pattern of circulating let-7b in IS patients with large-vessel atherosclerosis (LA) was increased in contrast to what observed in patients with other stroke subtypes (i.e., small-vessel disease (SV), cardioembolic stroke (CE) and undetermined cause (UDN) groups). Follow-up evaluations revealed that all circulating miRNAs levels returned to the baseline 48 weeks after the episode. Moreover, based on the level of three miRNAs authors defined a specific clinical score system that was further correlated with functional status as evaluated with the modified Rankin Scale (mRS), suggesting that miR-30a, miR-126, and let-7b could be used as a reliable marker for the diagnosis of IS (See Table 1 and Table 2) [41]. It is particularly interesting that a modulation of miR-30 has also been reported in patients with acute myocardial infarction [42]. If on one hand this strengthens the hypothesis that miRNAs are “smart” biomarkers capable of reflecting the pathophysiological processes underlying CVS, on the other hand it undermines its usefulness as a disease-specific biomarker. 

In another study, two miRNAs selected based on literature search, namely let-7e and miR-338, were analyzed in serum from IS patients in three phases of the ischemic event (i.e., acute- 1–7 days, subacute- 8–14 days, recovered- over 15 days) and miRNA levels were compared with 51 healthy volunteers. In this study only let-7e expression levels were significantly higher at all time-points with significantly higher levels in IS patients at the acute stage. Moreover, let-7e level was correlated with serum high sensitivity C-reactive protein (hs-CRP) level (r = 0.67, *p* = 0.033). A prognostic role of both miRNAs failed to be proven as there was no correlation between miRNAs expression level and National Institutes of Health Stroke Scale (NIHSS) scores, which is a widely used tool that determine the severity of a stroke. However, serum let-7e showed a specificity up to 73.4% and a sensitivity of 82.8% in IS patients at the acute stage, whereas serum miR-338 in IS patients showed a specificity up to 53.2% and a sensitivity of 71.9% in the acute stage. Thus, authors suggested that let-7e expression in serum may serve as a useful noninvasive circulating biomarker for the acute stage of IS [43]. 

Huang et al. sought to evaluate another let-7 family member, let-7e-5p, in two independent case-control IS populations. The results showed that the expression level of let-7e-5p was significantly higher in IS patients than in control subjects. Logistic regression analysis revealed that let-7e-5p expression was associated with an increased risk of IS (adjusted OR, 1.89; 95% CI, 1.61~2.21; *p* < 0.001). Also, diagnostic accuracy of acute phase specific miRNAs were tested through calculating the area under curve (AUC) of receiver operating characteristic (ROC) curves. The addition let-7e-5p to the traditional risk factor model improved the diagnostic potential to an AUC of 0.82 (95% CI, 0.78~0.85). In order to find targets of let-7e both bioinformatics and target gene expression analysis were performed. It showed that let-7e-5p expression was negatively correlated with several genes (ATF2, CASP3, FGFR2, NLK, PTPN7, RASGRP1, and TGFBR1). Therefore, the study showed that let-7e-5p expression is significantly higher in IS patients and is associated with the occurrence of IS. Moreover, authors suggested that let-7e-5p may be involved in the pathogenesis of IS by regulating CASP3 and NLK expression, as two genes enriched in the MAPK signaling pathway [44]. 

Gong et al. performed study in order to determine prognostic value of several let-7 family members (let-7a, let-7b, let-7c, let-7d, let-7e, let-7f, let-7g, miR-98) and relation to massive cerebral infarction (MCI) within first 48 h from the onset of symptoms. In their report, the expression of let-7f was down regulated in IS with MCI in comparison to healthy controls and IS without MCI, and up regulated in group without MCI at baseline, i.e., 48 h. When comparing relative expression of let-7f between groups with and without hemorrhagic transformation (HT), authors found up-regulation of let-7f in the MCI without HT after two weeks from the baseline. However, when compared MCI with and without HT significant up-regulation of let-7f was found in the first group after two weeks. It is worth to mention that the level of hs-CRP was negatively correlated with the relative expression of let-7f in the MCI group. Another important finding of the study showed that the expression level of let-7f in the MCI without HT is positively correlated with patients status based on Glasgow Coma Scale score and negatively with hs-CRP concentration (r = −0.88, *p* < 0.0001). Also in this study target gene expression analysis was performed and the relative expression of let-7f was negatively correlated with interleukin-6 (IL-6) expression in the MCI without HT (48 h and 2 weeks) (r = −0.40, *p* < 0.001), but not in the MCI with HT group, which may suggest that the downregulation of let-7f expression in patients with MCI without HT may induce inflammation [45]. The large involvement of let-7 with cerebrovascular disease, as proven by multiple evidences from independent studies is particular interesting. In fact, let-7 expression and availability is influenced by a circular noncoding RNA named CircPVT1, involved in the modulation of cell senescence [46].

Leung et al. investigated and compared plasma concentrations of miR-124-3p and miR-16 as diagnostic markers in acute stroke. Ninety-three patients with IS, 19 patients with hemorrhagic stroke, and 23 healthy controls enrolled in the study. Plasma concentrations of miRNAs were determined by RT- PCR. Median plasma 124-3p concentrations taken within 24 h of symptom onset were higher in hemorrhagic stroke patients than that in IS patients, while median miR-16 concentration in IS patients were higher than that in hemorrhagic stroke patients. Authors concluded that both miR-124-3p and miR-16 are diagnostic markers to discriminate hemorrhagic stroke and IS [47]. Another study aimed to investigate circulating miRNAs namely miR-15a, miR-16, and miR-17-5p [48]. The selection of specific miRNA was based on assumption that miR-15a and miR-16 are increased in the serum of patients with ischemic events and miR-17-5p may be a critical factor in post-stroke adult neurogenesis [49,50,51,52]. In this study, Wu et al. evaluated the utility of these three miRNAs in peripheral blood of 106 IS patients and 120 healthy controls as IS serum biomarkers for diagnostic value. Serum levels of miR-15a, miR-16, and miR-17-5p were significantly higher in IS patients compared to control subjects. Serum miR-15a levels showed a significant positive correlation with age. Besides, there was a strong negative correlation between serum miR-16 levels and high-density lipoprotein (HDL) and apolipoprotein A1 (ApoA1). Moreover, in multivariate logistic regression model, it was found that serum miR-17-5p level was a significant and independent predictor for IS (OR 3.968; CI 95% 1.001–14.29, *p* = 0.035). Also, ROC analysis showed that selected miRNAs were useful IS biomarkers (See Table 2) [48]. In line with these findings, miR-16 was recently associated to peripheral ischemia and was suggested as a potential mediator of remote vascular remodeling [19]. Hence, it could reflect systemic vascular dysfunction, rather than specific and acute localization of ischemic disease.

Jin and Xing evaluated 28 miRNAs selected based on previous publications that were described to possess pro-angiogenic or anti-angiogenic properties. Selected miRNAs were evaluated in plasma samples of IS patients and controls taken within 24 h after ischemic events. In the exploring stage performed in 10 patients and 10 controls, 11 differentially expressed miRNAs (DEM) were identified and included into the validating stage. In the second stage, in order to validate the significantly expressed miRNAs, 106 IS patients and 110 controls were analyzed. The expression of miR-126, miR-130a, and miR-378 in plasma decreased in IS patients; however, miR-222, miR-218, and miR-185 plasma levels were increased. At logistic regression analysis they found that miR-126 (OR 0.840; CI 95% 0.766–0.9220), miR-130a (OR 0.885; CI 95% 0.827–0.948), miR-222 (OR 1.064; CI 95% 1.004–1.126), miR-218 (OR 1.138; CI 95% 1.036–1.250), and miR-185 (OR 1.099; CI 95% 1.003–1.205) were independent predictor factors for IS. Moreover, the combined analysis of these five DEMs demonstrated a good diagnostic performance for IS with sensitivity of 87.7%, and specificity of 54.5%. Additionally, miR-126, miR-378, and miR-101 were negatively associated, and miR-222, while miR-218, miR-206 positively associated with NIHSS score what could be used for disease severity management of IS [53].

So far, several studies showed the importance of miR-145 in stroke. In one of the first study, circulating miR-145 was evaluated in 32 IS patients and compared to 14 control participants. The results showed significant up-regulation of circulating miR-145 expression and non-significant downregulation after one month in IS patients (*N* = 11). Authors suggested that miR-145 might be a desirable biomarker in IS [54].

In the next study nine previously reported stroke associated miRNAs (miR-21, miR-23a, miR-29b, miR-124, miR-145, miR-210, miR-221, miR-223 and miR-483-5p) were screened both in 146 IS group and 96 control subjects. In this validation process only miR-145 was significantly up-regulated, but miR-23a and miR-221 were significantly downregulated. Serum miR-23a and miR-221 were moderatly negatively correlated with plasma hs-CRP. Moreover, a strong positive correlation existed between serum miR-145 and hs-CRP (r = 0.6713), and a moderate correlation existed between serum miR-145 and IL-6 (r = 0.5896). Interestingly, miR-145 level was positively correlated with infarct volume and NIHSS scores (r = 0.6249 and r = 0.6288, respectively). Finally, the prediction value using both hs-CRP and miR-145 was significantly higher than for hs-CRP alone (See Table 2). In 49 IS patients long term follow-up lasting 2 years was performed and showed that the expression of miR-145 was highest within the acute phase (1 to 7 days) of stroke, but decreased within the recovery phase (1 month, 6 months, and 2 years) in the serum of patients [55]. The abovementioned role of miR-145 was confirmed through bioinformatics analyses (Gene Ontology and Kyoto Encyclopedia of Genes and Genomes enrichment) that included two mRNA and 1 miRNA microarray expression profile data from the Gene Expression Omnibus database. Based on this analysis two miRNAs, namely miR-145 and miR-122, may represent potential biomarkers in IS. Also, three novel miRNAs (miR-99b, miR-542-3p, and miR-455-5p) were deregulated what may suggest their roles in the pathological processes of IS [56].

Contrary to recent studies, Tsai et al. did not find significant modulation of miR-145 in IS. However, they found that miR-21 level was significantly higher, but miR-221 was significantly lower compared to healthy controls. MiR-145 was excluded from further analysis, as there were no significant differences between groups. At multivariable logistic regression analysis both increased miR-21 level (OR 6.16; 95% CI 2.82, 14.64) and decreased miR-221 level (OR 10.38; 95%CI 4.52, 26.45) were associated with increased risk of stroke when added to classical risk factors like age, sex, diabetes, hypertension, smoking, and hyperlipidemia. Also ROC analysis showed that adding both miR-21 and miR-221 into the model substantially improved AUC value for IS prediction. Hence, the authors suggested that miR-21 and miR-221 can be novel biomarkers for stroke, but not miR-145 [57]. 

Also, Zhou and Zhang identified decreased plasma miR-21 and miR-24 levels in 68 IS patients compared with 21 healthy controls. Moreover, miR-21 expressions correlated with miR-24 level, and both miRNAs negatively correlated with early outcome of IS based on NIHHS score (r = −0.703, *p* < 0.05 for miR-21; r = −0.694, *p* < 0.05 for miR-24) [58]. 

Previous study analyzed the changes of miR-223 levels in 75 healthy control samples and compared to 79 IS patients within 72 h from the onset of symptoms. It was found that miR-223 expression correlates with stroke subtype (i.e., LA and SA subtype) and negatively correlates with NIHSS scores, but the correlations with infarct volume and insulin-like growth factor 1 (IGF-1) levels were low [59]. Beside miRNA studies in circulating blood miR-223 was detect also in exosomes. Exosomes are 30–100 nm vesicles that cells secrete into extracellular space when multivesicular bodies fuse with cell membrane. MiR-223 was shown to be one of the most highly expressed miRNAs in plasma exosomes of healthy human and results about its function in ischemia injury are inconsistent [60]. Chen et al. found that the level of miR-223 in circulating exosomes was elevated after onset of IS, and exosomal miR-223 expression was positively correlated to NIHSS score. Moreover, stroke patients with poor outcomes based on mRS inclined to have a greater exosomal miR-223 expression. Therefore, increased exosomal miRNA-223 possess both diagnostic and prognostic value for IS [61]. 

In another study serum exosomal miRNAs, namely miR-9 and miR-124, were evaluated in 65 IS patients and compared with 66 non-stroke volunteers in order to explore their diagnostic and prognostic value. The team found that both miR-9 and miR-124 levels were increased in IS patients. Moreover, both miRNAs expression were positively correlated with NIHSS score (r = 0.7126 and 0.6825 respectively, *p* < 0.01), infarct volume evaluated by magnetic resonance imaging (MRI) (r = 0.6768 and 0.6312, respectively, *p* < 0.01) and serum IL-6 concentration (r = 0.6980 and 0.6550, respectively, *p* < 0.01). Finally, diagnostic value for IS of exosomal miR-9 and miR-124 was confirmed in ROC analysis [62]. Interestingly, another small study done by Liu et al. showed that serum expression of miR-124 was downregulated within the first 24 h after the ischemic event, but there were no differences in miR-9 and miR-219 levels between 31 IS and 11 healthy controls. Also, only miR-124 and miR-9 were negatively correlated with infarct lesion volume (r = −0.423, *p* = 0.022; and r = −0.608, *p* < 0.001), but not with NIHSS score. Besides, a significant negative correlation existed between plasma hs-CRP levels and serum miR-124 and miR-9 levels (r = −0.421, *p* = 0.023; and r = −0.511, *p* = 0.004) [63]. 

In the latest study, 17 previously reported stroke-associated miRNAs were measured in serum using RT-qPCR. Researchers first evaluated miRNA levels in randomly selected 30 IS patients compared with 30 control participants. MiR-21, miR-145, miR-29b, and miR-146b were significantly increased but miR-23a and miR-221 levels were decreased within 24 h after stroke onset compared with the control group. Further verification was done for miR-21, miR-23a, miR-29b, miR-145, miR-146b, and miR-221 with a larger number of patients. MiR-146b was significantly upregulated in the serum of 128 IS patients compared with 102 control participants. In addition to this, positive correlation was found between upregulated serum miR-146b level and plasma hs-CRP, infarct volume and NIHSS score, and serum IL-6 of patients. Importantly, the combination of plasma hs-CRP and serum miR-146b gained a better sensitivity/specificity for prediction of IS (AUC from 0.782 to 0.863). Besides, it was found that decreased miR-221 level negatively correlated with plasma hs-CRP level but not serum IL-6 level in IS patients. Authors suggested that upregulated serum miR-146b might be a potential diagnostic biomarker for IS evaluation [64].

### 2.2. MiRNA Profiling and RNA Sequencing Strategy

MiRNA microarrays are commonly used for miRNA expression profiling analysis. They have advantages and disadvantages comparison to RT-qPCR technologies. First of all, they allow us a powerful high-throughput profiling for a genome-wide miRNA profiling assay and the protocol can be easily standardized. Despite, there are some major disadvantages associated with miRNA microarrays. For example, generally a large amount of high-quality RNA samples are needed for the microarray experiments, which is often a major challenge for miRNA expression analysis of clinical samples. Moreover, lower miRNA detection sensitivity and specificity is the main limitation of microarray analysis compared to RT-qPCR analysis [65]. Beside miRNA microarrays, the next-generation-sequencing technology has quickly emerged as the preferred platform for studying circulating miRNA profiling. Comparison to microarray technology RNA-sequencing technology has higher sensitivity and specificity, however high cost is a practical consideration for researchers [66].

In one of the first study using microarray strategy Jickling et al. identified candidate miRNAs that could serve as a diagnostic tool in 24 IS patients. In a group with IS six miRNAs, expression was increased (i.e., miR-122, miR-148a, let-7i, miR-19a, miR-320d, miR-4429), and two miRNAs, expression was decreased (miR-363 and miR-487b) in comparison to healthy controls. By using gene target database, they also evaluated potential gene targets for selected miRNAs and they found two main pathways which are regulated by these miRNAs, i.e., the nuclear factor-κB (NF-κB) and Toll-like receptor signaling pathways, the pathways, which are involved in immune activation, leukocyte extravasation and thrombosis [67].

Using screening technique Li et al. discovered 115 miRNAs that were differentially expressed in IS patients compared to controls. For the further analysis they selected based on well-defined criteria and literature search 13 miRNAs. In order to validate the initial results they performed RT-qPCR of selected miRNAs and found that expression of 8 miRNAs (miR-32-3p, miR-106b-5p, miR-423-5p, miR-451a, miR-1246, miR-1299, miR-3149, and miR-4739) significantly increased, and the expression of 5 miRNAs (miR-224-3p, miR-377-5p, miR-518b, miR-532-5p, and miR-1913) significantly decreased in the serum of IS patients. According to functional assays only upregulated miR-32-3p, miR-106b-5p, and miR-1246, and downregulated miR-532-5p in IS serum might play a vital role in the pathogenesis of IS. Moreover, through bioinformatic analysis, they found that stroke-related genes vascular endothelial growth factor-A (VEGFA), myeloid cell leukemia-1 (Mcl-1), and superoxide dismutase 2 (SOD2) might be the targets of miR-106b, therefore authors suggested that miR-106b may affect multiple pathways such as apoptosis, oxidation, angiogenesis, and neurogenesis in IS [68,69,70,71]. 

MiRNA profiling strategy in the whole blood samples was proposed to analyze the utility of miRNA for the disease progression and stroke subtype evaluation in young patients with IS. This strategy showed that 157 miRNAs were differentially regulated across stroke samples. In total, 138 miRNAs were upregulated, and 19 miRNAs were downregulated. Interestingly, different miRNAs expression was observed between stroke subtypes groups, i.e., 8 miRNAs were downregulated (hsa-let-7f, miR-126, -1259, -142-3p, -15b, -186, -519e, and -768-5p) and 17 miRNAs were upregulated (hsa-let-7e, miR-1184, -1246, -1261, -1275, -1285, -1290, -181a, -25*, -513a-5p, -550, -602, -665, -891a, -933, -939, and -923) across subtypes of stroke. Further analysis showed also the utility of miRNA profiling in prognosis [72]. 

**Figure 1:**
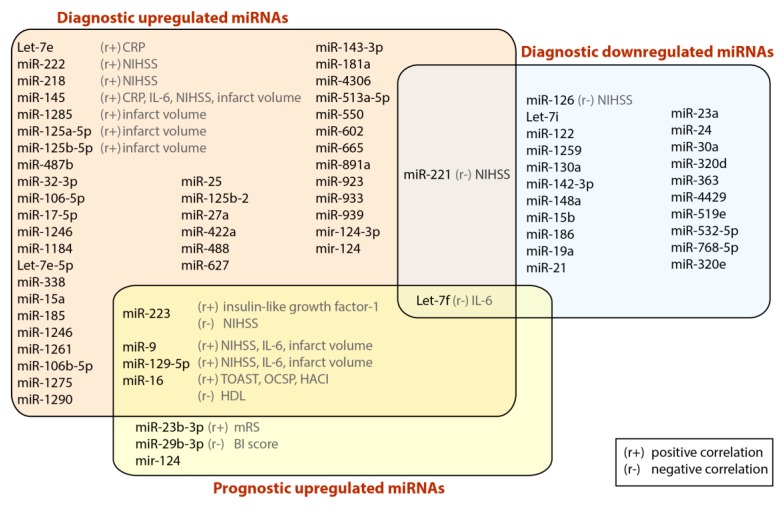
Regulation of diagnostic and prognostic miRNAs serving as biomarkers in ischemic stroke, based on human studies. Abbreviation: miRNA, microRNA; CRP, C-reactive protein; HDL, high density lipoprotein; NIHSS, National Institutes of Health Stroke Scale; IL-6, interleukin 6; TOAST, Trial of Org 10,172 in Acute Stroke Treatment; OCSP, Oxfordshire Community Stroke Project; HACI, hyperacute cerebral infarction; mRS, Modified Rankin Scale; BI, Barthel index. Refs: [41,43,44,45,47,48,53,55,57,58,61,62,67,68,72,73,74,75,76,78].

Profile of circulating miRNA was also evaluated by Sepramaniam et al. in three different cohorts that contained 169 patients with IS. In total 314 miRNAs were detected upon profiling. In the initial step of the analysis they found 105 different miRNAs that were deregulated in stroke cases. Among the 105 miRNAs, 58 were downregulated while 47 were upregulated. Moreover, further analysis help to significantly distinguish the stroke etiology i.e., LA, CE and SV based on 32 miRNAs (let-7a, let-7d*, let-7g, let-7i, miR-126, -130a, -187*, -18a*, -20a, -22*, -26b, -30b, -30c, -30e*, -320b, -320d, -324-5p, -331-3p, -340, -342-3p, -361-5p, -363, -422a, -423-3p, -501-5p, -502-3p, -505*, -574-3p, -675,-886-5p, -92a, and -93*), however detailed analysis was not provided in the study. Finally, the patients were segregated into acute and recovery phase and 26 miRNAs (let-7d*, miR-125b-2*, -1261, -1299, -130a, -1321, -208a, -22*, -23a, -27a*, -320b, -320d, -30c, -340, -422a, -423-3p, -488, -502-5p, -549a, -574-3p, -574-5p, -617, -627, -886-5p, -92a, and -93*) were unique for acute stroke and 16 miRNAs (let-7a, let-7g, miR-129-5p, -192-5p, -196a*, -26b, -30b, -30e*, -370, -381, -493*, -525-5p, -652, -920, -933, and -96) were unique for stroke patients at 7 days after the event (“recovered patients”). Diagnostic accuracy for acute phase of IS tested through calculating the AUC of ROC curves showed that 5 different miRNAs could serve as a potential biomarkers i.e., miR-125b-2*, -27a*, -422a, -488 and -627 [73] (See Figure 1). 

Profiling of miRNAs expression in plasma samples taken from IS patients was performed in 136 individuals with MRI evaluation on admission. In this population 76 patients had already ischemic changes detected with MRI (MRI+). Initial analysis revealed 120 miRNAs in MRI (−) group differentially expressed than in MRI (+) group and 69 miRNAs in MRI (+) in comparison to control group. Seventeen miRNAs were significantly changed (i.e., 10-fold) in plasma samples from both MRI (−) and (+) IS patients compared with control subjects and were further evaluated. However, only two miRNAs (hsa-miR-106b-5p and hsa-miR-4306) showed a gradient of increase, and only two miRNAs (hsa-miR-320e and hsa-miR-320d) showed a gradient of decrease from control patients to MRI(−) and MRI(+) IS patients. Also, ROC analysis performed on data from all IS patients using 4 miRNAs profile allowed to discriminate patients with IS from healthy controls. Thus, the authors showed that hsa-miR-106b-5p, hsa-miR-4306, hsa-miR-320e, and hsa-miR-320d might facilitate the diagnosis and clinical management of IS [74].

Another study aimed to identify specific circulating miRNAs that would facilitate the diagnosis of hyperacute cerebral infarction less than 6 h from the acute event, and validate their usefulness in follow-up up to 3 months after the onset of cerebral infarction. Forty patients with hyperacute cerebral infarction and 30 age-matched healthy volunteers were recruited in this study. Seven hyperacute cerebral infarction and four age-matched healthy volunteers were selected randomly for microarray analysis. Thirty-three patients and 23 controls were selected for RT-qPCR validation. In discovery phase microarray analysis showed 11 miRNAs that were upregulated (miR-140, miR-106b, miR-130a, miR-16, miR-223, miR-93, miR-484, miR-25, miR-130b, miR-107, and miR-151) and 1 miRNA that was downregulated (miR-4454), however in validation phase, only miR-16 was significantly different between patients and the control group. In multivariate logistic regression analysis only miR-16 (OR 1.669, 95% CI 1.071 ± 2.602, *p* = 0.024) was predictive for hyperacute cerebral infarction stroke. Moreover, miR-16 expression was significantly higher in the poor prognosis group than in the good prognosis group during follow-up and the diagnostic accuracy of miR-16 as a biomarker for was confirmed in ROC analysis [75].

In previously published research that consisted from three stages: discovery stage (20 IS patients and 20 healthy control subjects), validation stage (40 IS patients and 40 controls), and replication stage (200 IS patients and 100 controls). RNA sequencing was performed in order to study expression changes of circulating miRNAs. Out of 32 miRNAs, three miRNAs were upregulated in IS patients compared to both controls (i.e., miR-125a-5p, miR-125b-5p, and miR-143-3p) and transient ischemic attacks (TIA) patients. In long-term follow-up lasting up to 90 days both miR-125b-5p and miR-143-3p levels decreased, starting at day two, with no significant difference compared to controls. Expression levels of miR-125a-5p subsequently increased and stayed constantly elevated in comparison to control group. It is worth mentioning that in the studied population, different stroke subtypes were included, and miR-125a-5p, miR-125b-5p, and miR-143-3p were similar across etiological subgroups, i.e., patients with LA, CE, and stroke of undetermined etiology. Moreover, miR-125a-5p, miR-125b-5p, and miR-143-3p differentiated between controls and IS patients with an area AUC of 0.90 (sensitivity: 85.6%; specificity: 76.3%). Importantly, they found the expression levels of miR-125a-5p, miR-125b-5p, and miR-143-3p to be independent of infarct volume and stroke etiology. This finding emphasizes potential utility of these miRNAs as a broadly applicable diagnostic marker for IS. Based on another step of the experiment it was shown that the most important source of these miRNAs are platelets. Moreover, significantly higher concentration of miR-143-3p were found in extracellular microvesicles isolated from IS patients in comparison to healthy controls, but not for miR-125a-5p or miR-125b-5p [76]. 

One of the largest studies was done by Mick et al. They identify and validate ex-RNAs (extracellular noncoding RNAs) from plasma in participants of the Framingham Heart Study (FHS). The results demonstrate that when studied in a large observational cohort, miRNAs are significantly associated with stroke. In comparison to previously published studies there are some differences making this study highly valuable. First of all it was the largest, unbiased, community-based report of association between plasma circulating ex-RNAs (miRNAs, piRNAs, and snoRNAs) and stroke. Secondly as a method for ex-RNA discovery RNA sequencing was used that allowed to search not only for miRNAs, but also some other type of non-coding RNA like piRNAs, and snoRNAs. They selected the most abundantly expressed ex-RNAs by RNASeq (331 miRNAs, 97 piRNAs, and 43 snoRNAs) for RT-qPCR analysis in the entire FHS Offspring Cohort. Based on proposed strategy 3 miRNAs (miR-877-5p, miR-124-3p, and miR-320d) and one snoRNA (SNO1402) were independently associated with prevalent stroke. Moreover, two other miRNAs i.e., miR-656-3p and miR-941 were significantly associated with incident stroke risk adjusting for each other and potentially confounding clinical variables. However, such a strategy did not allow analysis of the relation between IS subtypes and miRNAs [77]. 

Wu et al. evaluated the expression patterns of specific miRNAs in TIA patients. In their study, 754 miRNAs were initially screened by the TaqMan Low Density Array (TLDA) in two pooled serum samples from 50 IS patients and 50 controls. After miRNA profiling, significantly changed miRNAs validated by RT-qPCR in the same cohort and further confirmed in another larger cohort including 177 IS, 81 TIA patients, and 42 controls. Consequently, TLDA screening showed that 71 miRNAs were upregulated, and 49 miRNAs were downregulated in IS patients. RT-qPCR validation confirmed that serum levels of miR-23b-3p, miR-29b-3p, miR-181a-5p, and miR-21–5p were markedly increased in IS patients. Strikingly, miR-23b-3p, miR-29b-3p, and miR-181a-5p were also significantly elevated in TIA patients. Logistic regression and ROC curve analyses showed that these changed miRNAs may function as predictive and discriminative biomarkers for IS and TIA. Thus, authors suggested that, their distinctive expression signatures may contribute to assessing neurological deficit severity of IS and subsequent stroke risk after TIA [78].

Summing up the above reported results, accumulating evidence indicates that circulating miRNAs might be used as an innovative diagnostic and prognostic biomarker and potential novel therapeutic target through its potential roles in inflammatory processes. However, to better evaluate the diagnostic and prognostic role of miRNAs, more studies are required to investigate the intricate interactions between the miRNAs and their target genes in IS. Results of the studies presented in this review should be interpreted with consciousness. As we discussed above studies found controversy findings. The discrepancy of the results might be the reason of demographic differences between populations, heterogeneity of populations, various cohort sizes and study designs. It is necessary to conduct further studies to validate the current hypotheses and closely determine the association between various miRNAs and their contribution to cardiovascular diseases development. 

## 3. Future Perspectives for Using miRNAs in Diagnosis and Prognosis in Ischemic Stroke

The use of circulating miRNAs as disease biomarkers in stroke is a very attractive and promising concept. In fact, they might offer several advantages over traditional disease biomarkers: (i) they can reflect specific cellular pathophysiological alterations; (ii) experimental evidence suggests that they could potentially indicate the specific etiology of stroke; (iii) since specific miRNAs showed significant modulation well before the development of the acute and irreversible cerebral damage, they could potentially allow early stroke diagnosis and/or the identification of subjects at risk before they develop an acute stroke.

Nevertheless, a number of challenges still need to be addressed before circulating miRNAs could enter the clinical arena: (a) only few studies have tested their clinical usefulness, most of which are quite small and present methodological flaws; (b) the large variation observed for miRNAs within the same patients group could make the identification of diagnostic cutoffs particularly challenging; (c) most of the available studies that evaluated the clinical usefulness of circulating miRNAs in stroke were performed in Asian populations and their results could therefore not completely apply to other ethnicities; (d) most studies did not assess the impact of other clinical variables on the prognostic potential, which could be additive in some cases and thus potentially helpful if used to correct/adjust biomarker values; (e) finally, the current standard method for the reliable measurement of circulating miRNAs is RT-PCR, which presents some limitations for the use as clinical biomarker in the setting of an acute disease. Although several alternative methodological approaches are being tested, a reliable, fast and cheaper analytical methodology has not been established, yet.

## 4. Bioinformatics Analysis

We performed bioinformatics analysis in order to identify the most commonly regulated circulating miRNA among the selected studies that are involved in inflammation, blood coagulation, and platelet activation. The miRNAs list was narrowed down by searching human studies literature. R programming was used to build a miRNA-Gene-Network based on the interactions of the miRNAs and target genes and their functions. TargetScan and MirTarBase databases were used to predict the target genes of the miRNAs [79,80]. To identify the genes associated with inflammation process; platelet activation; blood coagulation we performed a screening of the Gene Ontology (GO) terms for the presence of the key words using the biomaRt package in R [81]. Key words used for screening the GO terms are given in the Supplementary Materials (See Table S1). Electronic database Pubmed and Scopus was searched between December 2017 and March 2018, and original studies were reviewed to evaluate the potential diagnostic or/and prognostic role of circulating miRNAs associated with IS. Review articles and meta-analyses were also investigated, and their secondary references were examined for possible inclusion. Our search was limited to human studies only and did not exclude studies on the basis of ethnicity of study participants. Search terms comprised of the following search syntax: “Search (“micrornas” [MeSH Terms] OR “mir” [MeSH Terms] OR “mirna” [MeSH Terms] OR “circulating miRNA” [MeSH Terms] OR “circulating microRNA” [MeSH Terms]) AND (“ischemic stroke” [MeSH Terms] OR “stroke” [All Fields]) AND (“diagnostic” [MeSH Terms] OR “diagnosis” [All Fields]) OR (“prognostic” [MeSH Terms] OR “prognosis” [All Fields])” Filters: Humans. A total of 52 records were identified after duplicates removal. Titles and abstracts were screened by two independent operators, with exclusion of 27 records for any of the following reasons: (a) they were not related to the specific research question (*n* = 11); (b) they did not present original data (*n* = 14); or they were not human studies (*n* = 2). Finally, 25 articles were selected to be used in this review.

**Figure 2:**
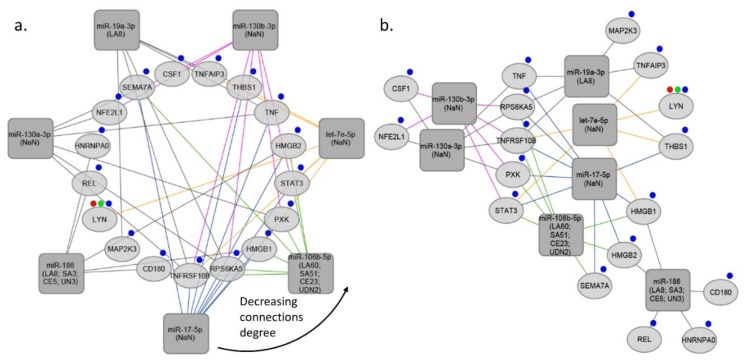
miRNA-target gene networks based on inflammatory response. (**a**) Inflammatory response-network sorted by the degree of connections, (**b**) Inflammatory response-interaction network. The rectangles indicate the stroke type miRNAs, the ellipses indicate target genes. Red, green and blue marks represent specific GO process-blood coagulation, platelet activation, and inflammation process, respectively. LA, large artery stroke; SA, small artery stroke; UDN, stroke due to undetermined cause; CE, cardioembolic stroke; NaN, no data.

**Figure 3:**
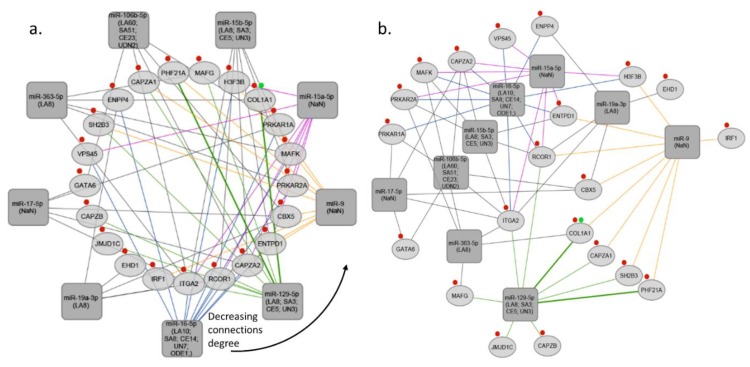
miRNA-target gene networks based on blood coagulation. (**a**) Blood coagulation- network sorted by the degree of connections, (**b**) Blood coagulation-interaction network. The rectangles indicate the stroke type miRNAs, the ellipses indicate target genes. Red and green marks represent specific GO process-blood coagulation and platelet activation, respectively. LA, large artery stroke; SA, small artery stroke; UDN, stroke due to undetermined cause; CE, cardioembolic stroke; NaN, no data; ODE, other determined etiology; UN, determined cause.

**Figure 4:**
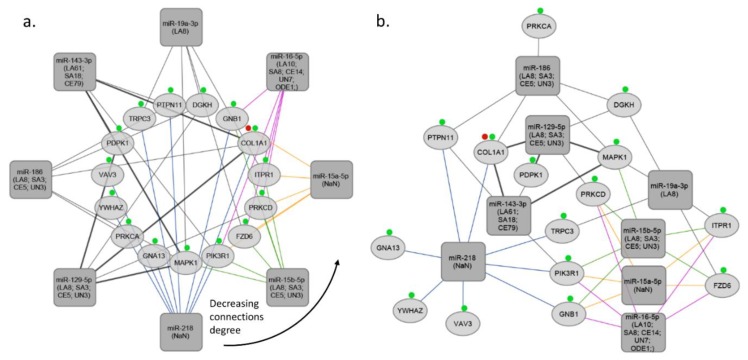
miRNA-target gene networks based on platelet activation. (**a**) Platelet activation-network sorted by the degree of connections, (**b**) Platelet activation-interaction network. The rectangles indicate the stroke type miRNAs, the ellipses indicate target genes. Red and green marks represent specific GO process-blood coagulation and platelet activation, respectively. LA, large artery stroke; SA, small artery stroke; UDN, stroke due to undetermined cause; CE, cardioembolic stroke; NaN, no data; ODE, other determined etiology; UN, determined cause.

MiRNA-target interaction network was constructed using R programming and visualized using Cytoscape software v 3.6.1 [82]. miRNA-target interactions used for constructing the network were obtained from TargetScan and MirTarBase databases. In the next step we selected miRNAs targeting at least 5 genes associated with specific GO process (inflammation process; platelet activation; blood coagulation), and target genes regulated by at least 5 miRNAs (See Figure S1b). In the third step we subsetted miRNAs regulating at least 5 target genes from the network created in the first step. Final networks were created by using circular layout and direct force layout (See Figure S1c). Circular networks were sorted by the degree of connections between miRNAs and their targeted genes. It enabled to retrieve top miRNAs and top shared targets involved in analyzed GO process (See Figure 2a, Figure 3a, and Figure 4a). To retrieve gene–gene interactions between our top targets we used interactome datasets from String app v 1.2.2 for Cytoscape [83].

The pathophysiology of IS is related with inflammation, blood coagulation process and platelet activation. In course of thromboembolic stroke, activated platelets orchestrate a thrombo-inflammatory cascade by promoting thrombus formation and growth, activating leukocytes, and potentiating cerebral endothelium injury. In line with this aim, we focused on miRNAs involved in inflammation, blood coagulation, and platelet activation in our bioinformatics analysis in order to identify to account for all potential pathophysiological mechanisms underlying the development of IS. In fact, the study that were selected included different subtypes of IS, such as thromboembolic, LA, SA, and UDN. We adopted such broad criteria is the intention to identify a pattern of possibly few miRNAs that could help to recognize the different underlying etiologies and/or main pathophysiological mechanisms. Using a similar approach, we were recently able to identify as few as two circulating miRNAs to be used for the differential diagnosis of heart failure of ischemic origin from other forms of non-ischemic origin [22].

**Figure 5:**
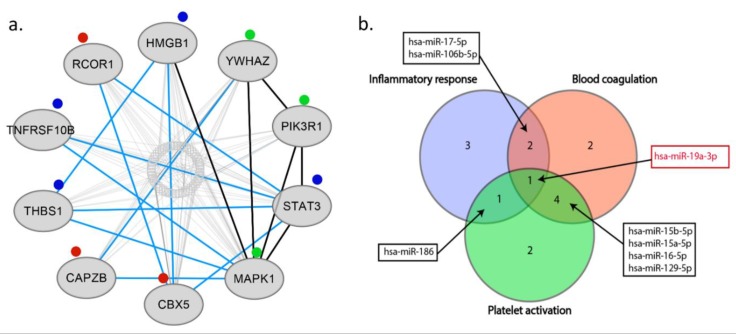
Most important genes targeted by miRNAs and overlapped miRNAs in GO process in IS. (**a**) Top ten genes targeted by miRNAs associated with IS. The circle in the middle indicates the stroke type miRNAs, the ellipses indicate target genes. Red, green and blue marks represent specific GO process-blood coagulation, platelet activation, and inflammation process, respectively. Black edges represent the high confidence connections between genes, blue edges represent low confidence connections. (**b**) Overlapped miRNAs in inflammatory response, blood coagulation, and platelet activation. 2 common miRNAs observed in inflammatory response and blood coagulation namely, miR-17-5p miR-106b-5p. 1 common miRNA observed in inflammatory response and platelet activation namely, miR-186. 4 common miRNAs observed in blood coagulation and platelet activation namely, miR-15b-5p, miR-15a-5p, miR-16-5p, miR-129-5p. 1 common miRNA observed in inflammatory response, blood coagulation and platelet activation namely, miR-19a-3p. miR, microRNA.

Our analysis showed that 2 common miRNAs namely, miR-17-5p and miR-106b-5p may regulate both inflammatory response and blood coagulation. 1 common miRNA, which is miR-186 may regulate both inflammatory response and platelet activation. Addition to this, we found that, 4 common miRNAs namely, miR-15b-5p, miR-15a-5p, miR-16-5p, and miR-129-5p may regulate both blood coagulation and platelet activation. Interestingly, 1 common miRNA, miR-19a-3p was found, that is involved in inflammatory response, blood coagulation, and platelet activation (See Figure 5b). Thus, we found that miR-19a-3p can be the crucial miRNA which regulates all of these three processes. MiR-19a is a member of the miR-17-92 cluster, and its importance was shown in breast cancer cells [84]. Besides, it was found that members of miR-17-92 cluster are involved in coronary artery disease and vascular functions, including ischemia responses and angiogenesis [85,86,87]. Liu et al. investigated the function of the miR17-92 cluster in adult neural progenitor cells after experimental stroke. They found that stroke substantially up-regulated miR17-92 cluster expression in neural progenitor cells of the adult mouse and inhibition of individual members of the miR17-92 cluster, such as miR-19a, suppressed cell proliferation and increased apoptosis [52]. As we discussed, Jinkling et al. demonstrate that, low circulating miR-19a levels in the blood were found in patients with IS compared to controls [67]. This can be the reason of potential cell protective effect of miR-19a in ischemia. It is worth underlining that only two studies out of 25 analyzed miR-19a-3p, and the only study which used miRNA profiling was found the significant relation with IS. There are several reasons that could help to understand such a discrepancy; (i) different inclusion and exclusion criteria implemented in each study, (ii) differences in population origin, (ii) lack of homogeneity of IS population due to inclusion of different IS subtypes. These weak points should be considered, and better-designed studies should confirm the actual biological role and clinical usefulness of miR-19a-3p in IS. Additionally, in our analysis we demonstrated ten the most significant genes targeted by miRNAs associated with blood coagulation, platelet activation, and inflammation process in IS, namely HMGB1, YWHAZ, PIK3R1, STAT3, MAPK1, CBX5, CAPZB, THBS1, TNFRSF10B, RCOR1 (High-mobility group box 1- HMGB1; tyrosine 3 monooxygenase/tryptophan 5 monooxygenase activating protein, zeta polypeptide- YWHAZ; phosphoinositide-3-kinase regulatory subunit 1- PIK3R1; signal transducers and activators of transcription 3- STAT3; mitogen-activated protein kinase 1- MAPK1; chromobox homolog 5- CBX5; capping actin protein of muscle Z-line subunit beta- CAPZB; thrombospondin-1- THBS1; TNF receptor superfamily member 10b- TNFRSF10B; REST corepresor 1- RCOR1) (See Figure 5a). 

HMGB1 is released from necrotic brain tissue and its differential redox forms attract and activate immune cells after ischemic brain injury. Its concentrations correlate with disease severity and outcome after brain injury [88]. Our results support the importance of HMGB in IS. The role of YWHAZ gene was shown in several neurodegenerative diseases such as, Huntington’s disease, Alzheimer’s disease, and amyotrophic lateral sclerosis [89,90,91]. As we found in our bioinformatic analysis a similar study showed the importance of YWHAZ gene in CE [92]. Besides, the role of PIK3R1 gene in stroke is yet to be published. On the other hand, one study showed that PIK3R1 is the target of miR-221 in endothelial progenitor cells (EPCs). EPCs assist angiogenesis and have been linked to ischemia-related disorders, including coronary artery disease [93]. Abovementioned, human studies showed that, miR-221 is a potential diagnostic biomarker in IS, thus the role of PIK3R1 and relation with miR-221 in IS should be confirmed by future studies [55,57,64]. As we showed the relation of STAT3 with IS in our analysis, lately Liang et al. reviewed several significant cerebral ischemic and HS treatments that target the STAT3 signaling pathway, including pharmacological and physical therapies [94]. Additionally, Bam et al. investigated miRNA profile in the peripheral blood mononuclear cells of IS patients. Further, they performed bioinformatic analysis and they showed that pro-inflammatory genes like STAT3, IL12A, and IL12B are some of the highly predicted targets for the dysregulated miRNAs [95]. Moreover, studies showed that MAPK can be an important regulator in ischemic and hemorrhagic cerebral vascular disease and raising the possibility that it might be a drug discovery target for stroke. In our bioinformatics analysis we confirmed previous results that showed the importance of MAPK1 gene and its signaling pathway in IS [44,68,96]. CBX5 is a member of the heterochromatin protein 1 (HP1) family, HPs are closely related to the development of major vascular injuries, such as atherosclerosis and hypertension. To the best of our knowledge, only one study showed that association of serum levels of antibodies against CBX5 could potentially represent useful tools for the diagnosis of TIA and predicting the onset of acute-phase of cerebral infarction [97]. In our analysis we showed that CBX5 can be related with IS targeted by miRNAs. THBS1 is a potent regulator of angiogenesis and previous studies showed the importance of THBS1 in stroke [98,99]. Gao et al. showed that plasma THBS1 protein concentrations are elevated and are highly associated with long-term outcome of IS [98]. Based on our bioinformatic analysis we also demonstrated the relation between THBS1 gene and IS targeted by miRNAs. It was shown that decreased cardiac CAPZ protein protects hearts against acute ischemia-reperfusion injury [100]. However, there is no published study describing the role of CAPZB in IS. Similarly, the role of RCOR1 and TNFRSF10B genes in stroke is yet to be published. In our analysis for the first time we found that CAPZB, RCOR1, and TNFRSF10B may have association with IS targeted by miRNAs.

## 5. Conclusions

The present article provides an up-to-date and comprehensive overview of all circulating miRNAs studied so far as diagnostic or prognostic markers in IS. As we deeply reviewed above, studies showed the impact of more than 1000 different miRNAs on IS, and their association with biochemical and hematological parameters in stroke patients. These studies clarify the value of miRNAs in IS the ischemic pathophysiological process and help us to better understand processes involved in IS pathophysiology. Through a bioinformatic analysis, we associated key biological function and signaling pathways related to the miRNAs with most prominent differential expression pattern in IS compared to controls. Using this approach, we found miR-19a-3p as the single miRNA involved in all three main biological processes selected for the bioinformatic analysis: inflammation, blood coagulation and platelet activation. Furthermore, results of bioinformatics analysis shows the following most significant genes targeted by miRNAs in IS: HMGB1, YWHAZ, PIK3R1, STAT3, MAPK1, CBX5, CAPZB, THBS1, TNFRSF10B, and RCOR1. Moreover, it is worth mentioning that CRP and IL-6 are the most common targets within the inflammatory ontology. This latter finding is particularly interesting, as both CRP and IL-6 are well-known prognostic markers in atherosclerotic vascular disease [101]. In addition, CRP might represent a key link between vascular inflammation and arterial thrombosis or platelet activation [102,103,104]. Finally, the finding that multiple miRNAs are correlated to inflammation supports the use of a panel of multiple miRNAs. 

In conclusion, since many known biomarkers related with IS are not disease specific and have been also associated with other types of brain injury, novel and specific diagnostic/prognostic biomarkers in IS are needed. In this context, circulating miRNAs are very promising biomarkers in stroke. Since specific miRNAs showed significant modulation well before the development of the acute and irreversible cerebral damage, they may potentially allow early stroke diagnosis and/or the identification of subjects at risk before they develop an acute stroke, however future studies are needed.

## Figures and Tables

**Table 1:** Human studies evaluating miRNAs as diagnostic/prognostic biomarkers in stroke.

Ref	miRNAs	Sampling/Sampling Time Point	Number of Stroke pts/Controls	Inclusion Criteria	Exclusion Criteria	Stroke Subtype	Prognostic or Diagnostic Value	Regulation of miRNAs	Correlation
Long et al. [41]	miR-30a, miR-126, Let-7b,	Plasma/24 h, 1 w, 4 w, 24 w, 48 w	197/50	First-ever stroke patients with cerebral infarction	Exclusion criteria included TIA, subarachnoid hemorrhage, embolic brain infarction, brain tumors, and cerebrovascular malformation, pulmonary fibrosis, endocrine and metabolic diseases (except type 2 diabetes), inflammatory and autoimmune diseases, and serious chronic diseases, for example, hepatic cirrhosis and renal failure. Cardioembolic stroke and documented atrial fibrillationwere also excluded from the study.	LA 51, SA 48, CE 50, UDN 48	Diagnostic value	MiR-30a and miR-126 were downregulated in 24 h, 1 w, 4 w, and 24 w. After 48 w miRNA levels increased to the baseline	No correlation was found between HDL, LDL, triglyceride, systolic and diastolic blood pressures, diabetes and smoking status and miRNAs.
Peng et al. [43]	miR-338, Let-7e	Serum and CSF/1–7 days (acute phase), 8–14 days (subacute phase), over 15 days (recovery)	72/51	Diagnosis of an initial episode of cerebral infarction based on clinical history and MRI results, ages ranging from 55 to 75 years, patient arrival at the hospital after 4.5 h but within 24 h after the event, NIHSS score of 4 to 15, and without hemorrhagic transformation.	Exclusion criteria for all enrolled patients included recurrent stroke, tumors, abnormal renal or liver function, infectious diseases, immune diseases, blood disorders, and psychiatric illness including depression and schizophrenia.	NA	Let-7e may be a biomarker in acute phase/had no prognostic value	Let-7e significantly higher at all time-points in serum. MiR-338 and Let-7e in CSF was upregulated in 8–14 days (subacute).	No correlation was found between NIHSS and Let-7e, correlation was found between CRP levels and Let-7e.
Huang et al. [44]	Let-7e-5p	Whole blood/24 h	Two groups: 44/44, 302/02	First-ever IS patients	Patients with a history of stroke, peripheral arterial occlusive disease or cancer were excluded from this study.	NA	Diagnostic value for Let-7e-5p	Let-7e-5p significantly higher with IS patients than controls	Negatively correlated with ATF2, CASP3, FGFR2, NLK, PTPN7, RASGRP1 and TGFBR1 genes.
Gong et al. [45]	Let-7f	Serum/after 48 h and 2 w	88/130	Selection criteria included age >18 years, within 48 h after stroke attack, based on CT or MRI, the patient had an infarct of at least 67% of the middle cerebral artery territory, with or without the additional infarction of the anterior or posterior cerebral artery on the same side.	Exclusion criteria included unconsciousness due to metabolic disturbances or medication, any sedation or surgery, a pre-stroke score on the mRS of more than 2; and the presence of a concurrent serious illness which may affect the patient’s outcome, such as severe cardiopulmonary complications.	NA	Prognostic value	Let-7f was downregulated in IS with MCI and upregulated in IS without MCI at 48 h.	Let-7f was negatively correlated with hs-CRP in IS MCI patients, also negatively correlated with IL-6 in MCI without HT
Leung et al. [47]	miR-124-3p and miR-16	Plasma/(≤6 h), (6–24 h)	93 IS + 19 HS/23	Patients aged 18 years old and above were included to the study, HS or IS confirmed by CT scan and/or MRI, who presented within 24 h of symptom onset.	NA	NA	Diagnostic value	MiR-124-3p levels were markedly higher in patients with HS patients compared to IS patients only in cases presenting early (≤6 h), increased miR-16 were found in patients with IS compared to those with HS in patients presenting late after symptom onset (6–24 h)	Plasma concentrations of miR-124-3p, but not miR-16, positively correlated with lesion volume on CT in HS patients; however, both plasma miR-124-3p and miR-16 did not correlate with lesion volume on MRI in IS patients.
Wu et al. [48]	miR-15a, miR-16, miR-17-5p	Serum/before treatment	106/120	The cohort included 55 men and 51 women with a mean age of 64.8 years (range, 39–88 years).	Symptoms indicative of subarachnoid hemorrhage, even if no imaging findings of hemorrhage were found on CT or MRI, intracranial hemorrhage, acute myocardial infarction, critical limb ischemia.	NA	Diagnostic value	MiR-15a, miR-16, and miR-17-5p were significantly higher in IS patients compared to control subjects	MiR-15a was significantly correlated with age, strong negative correlation between miR-16 levels and HDL and ApoA1 was found.
Jin F. and Xing J. [53]	miR-126, miR-17-5p, miR-17-3p, miR-18a, miR-19a, miR-20a, miR-19b-1, miR-92a, Let-7b, Let-7f, miR-130a, miR-210, miR-378, miR-296, miR-101, miR-221, miR-222, miR-328, miR-15b, miR-16, miR-26b, miR-27b, miR-218, miR-206, miR-338-3p, miR-497, miR-195a-3p, miR-185	Plasma/24h	106/110	Within 24 h post the onset of symptom, diagnosed with IS according to patient history, laboratory and neurological examination, CT scan, MRI, and/or MRA.	Patients with infection, renal or hepatic failure, hematological malignancies, solid tumors, immunosuppressive therapy, or treatment with thrombolytic therapy were excluded from the study.	NA	Diagnostic value, disease severity management	MiR-126 and miR-130a decreased in the IS patients while miR-222, miR-218, and miR-185 increased in the IS patients.	MiR-126, miR-378, miR-101 negatively, miR-222, miR-218, miR-206 were positively correlated with NIHSS
Gan et al. [54]	miR-145	Whole blood/NA/after one month second sampling (N = 11)	32/14	IS patients between the ages of 18 and 49 years were included. IS was confirmed by either MRI or CT imaging of the brain.	Excluded from the study were subjects with hemorrhage stroke.	NA	No diagnostic and prognostic value was found	Upregulation of miR-145	No correlation was found
Jia et al. [55]	miR-21, miR-23a, miR-29b, miR-124, miR-145, miR-210, miR-221, miR-223, miR-483-5p	Serum/24 h	146/96	Patients with IS within 24 h after symptom onset were included.	Exclusion criteria included under 18 years old, being on thrombolytic or anticoagulant therapies, intracerebral hemorrhage or hemorrhagic transformation, other complicating neurological or neuropsychological diseases, cancer, comorbidity with proinflammatory conditions and clinical signs of infection at any time during the study.	NA	Diagnostic value	MiR-145 was upregulated, miR-23a and miR-221 were significantly downregulated	Positive correlation between miR-145 and hs-CRP, IL-6, infarct volume and NIHSS scores was found, serum miR-23a and miR-221 were moderate negatively correlated with plasma hs-CRP
Tsai et al. [57]	miR-145, miR-21, miR-221	Serum/7 days	167/157	Patients with IS based on the World Health Organization criteria. The blood samples from the patients were taken within 7 days of the onset of stroke. Demographic data and histories of hypertension, diabetes, hypercholesterolemia and cigarette smoking were obtained from each study subject.	NA	NA	Diagnostic value	MiR-21 was downregulated, miR-221 was upregulated, no significance for miR-145 was found	MiR-21 expressions was correlated with miR-221 levels.
Zhou et al. [58]	miR-21, miR-24	Plasma/24 h	68/21	ACI participated included in the study. The diagnosis of ACI was conducted based on patient history, lab examination, neurological deficit, MRI and MRA results.	Patients with a history of tumor, immune disease, blood disease, acute infectious disease, renal or liver failure were excluded.	NA	Diagnostic value	MiR-21 and miR-24 were downregulated	MiR-21 expressions were positively correlated with miR-24 level, and negatively correlated with NIHHS score
Chen et al. [61]	miR-223	Exosomes/plasma/less than 72 h	50/33	Stroke patients included in the study. Demography feature, related previous history including hypertension, diabetes mellitus, hyperlipidemia, cardiopathy, associated laboratory test, and imaging information including blood glucose, blood lipid, electrocardiogram, cardiac ultrasonography, carotid artery ultrasonography, MRI, and MRA were also collected for analysis.	Exclusive criteria included recurrent stroke or stroke onset longer than 72 h, renal or liver failure, acute infectious disease, tumor, hematologic disease, and patients who are unable to cooperate with physical examination.	LA 25, SA 17, CE 8	Diagnostic and prognostic value	MiR-223 was upregulated	MiR-223 was positively correlated with NIHSS score
Wang et al. [59]	miR-223	Whole blood/less than 72 h	79/75	IS patients included in the study.	The exclusion criteria included recurrent stroke, intracranial tumor, multiple trauma, hematological system diseases, renal or liver failure, acute infectious diseases and other diseases affecting the hemogram. If the time from the onset of stroke symptoms to blood sample collection was longer than 72 h, the patient was excluded.	LA 37, SA 9, CE 5, UN 28	Diagnostic value	MiR-223 in IS patients were greatly increased compared to the control	MiRNA-223 was negatively correlated with NIHSS scores, plasma level of IGF-1 was positively correlated with that of miRNA-223
Ji et al. [62]	miR-9, miR-124	Exosomes/plasma/the mean time of enrollment blood draw was 16.5 h.	65/66	IS patients were recruited after either MRI or CT imaging of the brain.	Patients with intracerebral hemorrhage or unknown etiology were excluded.	NA	Diagnostic and prognostic value	MiR-9 and miR-124 in IS patients were increased compared to the control	The levels of both miR-9 and miR-124 were positively correlated with NIHSS scores, infarct volumes and serum concentrations of IL-6.
Liu et al. [63]	miR-9, miR-124, miR-219	Serum/24 h	31/11	Patients with IS 24 h after symptom onset were enrolled to the study.	Exclusion criteria were being under 18 years old, being on thrombolytic or anticoagulant therapies, intracerebral hemorrhage or hemorrhagic transformation, other complicating neurological or neuropsychological diseases, cancer, comorbidity with proinflammatory conditions, and clinical signs of infection at any time during the study.	NA	Inflammatory value	MiR-124 was downregulated	Both serum miR-124 and miR-9 levels within 24 h were negatively correlated with infarct volume and plasma hs-CRP levels. All three miRNAs were negatively correlated with MMP-9 levels.
Jickling et al. [67]	miR-122, miR-148a, Let-7i, miR-19a, miR-320d, miR-4429, miR-363, miR-487b	Whole blood/NA	24/24	Patients with IS were enrolled to the study. Stroke diagnosis restricted diffusion on brain MRI (positive DWI-MRI).	Patients with infection (current or within 2 weeks of stroke), immunosuppressive therapy, lymphoma, leukemia, or treatment with thrombolytic therapy were excluded from study.	LA 8, SA 8, CE 8	Diagnostic value	In patients with IS, miR-122, miR-148a, let-7i, miR-19a, miR-320d, miR-4429 were decreased and miR-363, miR-487b were increased compared to vascular risk factor controls.	MiRNAs may regulates NF-κB and toll-like receptor signaling pathways, which are involved in immune activation, leukocyte extravasation and thrombosis.
Li et al. [68]	In total 115 miRNAs were screened miR-32-3p, miR-106b-5p, miR-423-5p, miR-451a, miR-1246, miR-1299, miR-3149, miR-4739, miR-224-3p, miR-377-5p, miR-518b, miR-532-5p, miR-1913	Serum/24 h	117/82	IS patients (aged >45) within 24 h after stroke onset were enrolled to the study.	Exclusion criteria included other types of stroke (TIA, subarachnoid hemorrhage, brain tumors, and cerebrovascular malformation); severe systemic diseases, i.e., pulmonary fibrosis, endocrine, and metabolic diseases (except type 2 diabetes); inflammatory and autoimmune diseases; and serious chronic diseases, for example, hepatic cirrhosis and renal failure.	NA	Diagnostic value of upregulated miR-32-3p, miR-106b-5p, miR-1246, and downregulated miR-532-5p	MiR-32-3p, miR-106b-5p, miR-423-5p, miR-451a, miR-1246, miR-1299, miR-3149, miR-4739 were upregulated	MiR-106b may affect multiple pathways such as apoptosis, oxidation, angiogenesis, and neurogenesis in IS.
Tan et al. [72]	in total 157 miRNAs were screened hsa-let-7f, miR-126, -1259, -142-3p, -15b,-186, -519e, -768-5p hsa-let-7e, miR-1184, -1246, -1261, -1275, -1285, -1290, -181a, -25*, -513a-5p, -550, -602, -665, -891a, -933, -939, -923	Whole blood/within 6–18 months	19/5	Asian stroke patients between the ages of 18 to 49 were enrolled to the study. Blood samples collected from stroke patients within 6–18 months in time scale from the index stroke. IS was confirmed either with CT or MRI of the brain.	NA	LA 8, SA 3, CE 5, UN 3	Diagnostic value	In total 138 miRNAs were upregulated and in total 19 miRNAs were downregulated. hsa-let-7f, miR-126, -1259, -142-3p, -15b,-186, -519e, -768-5p were downregulated, hsa-let-7e, miR-1184, -1246, -1261, -1275, -1285, -1290, -181a, -25*, -513a-5p, -550, -602, -665, -891a, -933, -939, -923 were upregulated.	Among the upregulated miRNAs, the expression of miR-101, -106b, -130a, -144, -18a, -18b, -19a, -19b, -194, -22, -22, -29b, -29c and -363 were the highest for LA (mRS = 3) stroke sample and positively correlated to the profile observed for LA mRS > 2
Sepramaniam et al. [73]	In total 314 miRNAs were screened. hsa-let-7e, hsa-miR-125b-2*, hsa-miR-1261, hsa-miR-129-5p, hsa-miR-1321, hsa-miR-135b, hsa-miR-145, hsa-miR-184, hsa-miR-187*, hsa-miR-196a*, hsa-miR-198, hsa-miR-200b*, hsa-miR-210, hsa-miR-214, hsa-miR-220c, hsa-miR-25*, hsa-miR-602, hsa-miR-611, hsa-miR-617, hsa-miR-623, hsa-miR-627, hsa-miR-637, hsa-miR-638, hsa-miR-659, hsa-miR-668, hsa-miR-671-5p, hsa-miR-675, hsa-miR-920, hsa-miR-933, hsa-miR-943, hsa-miR-99a, hsa-let-7a, hsa-let-7b*, hsa-let-7c, hsa-let-7d*, hsa-let-7f, hsa-let-7g, hsa-let-7i, hsa-miR-106b*, hsa-miR-126, hsa-miR-1299, hsa-miR-130a, hsa-miR-151-5p, hsa-miR-18a*, hsa-miR-182, hsa-miR-183, hsa-miR-186, hsa-miR-192, hsa-miR-20a, hsa-miR-208a, hsa-miR-22*, hsa-miR-500, hsa-miR-500*, hsa-miR-501-5p, hsa-miR-502-5p, hsa-miR-502-3p, hsa-miR-505*, hsa-miR-532-5p, hsa-miR-574-5p, hsa-miR-574-3p, hsa-miR-576-5p, hsa-miR-625, hsa-miR-629, hsa-miR-652, hsa-miR-7, hsa-miR-886-5p, hsa-miR-92a, hsa-miR-93*, hsa-miR-96	Whole blood/24 h, 48 h, 7 days, from 2 months to 2 years from stroke onset	169/118	Patients with IS were enrolled to the study. IS was confirmed through either MRI or CT imaging of the brain.	NA	NA	Diagnostic value, potential diagnostic biomarkers; miR-125b-2*, -27a*, -422a, -488 and -627	Among the significant 105 miRNAs, 58 were downregulated while 47 were upregulated. Upregulated miRNAs: hsa-let-7e, hsa-miR-125b-2*, hsa-miR-1261, hsa-miR-129-5p, hsa-miR-1321, hsa-miR-135b, hsa-miR-145, hsa-miR-184, hsa-miR-187*, hsa-miR-196a*, hsa-miR-198, hsa-miR-200b*, hsa-miR-210, hsa-miR-214, hsa-miR-220c, hsa-miR-25*, hsa-miR-602, hsa-miR-611, hsa-miR-617, hsa-miR-623, hsa-miR-627, hsa-miR-637, hsa-miR-638, hsa-miR-659, hsa-miR-668, hsa-miR-671-5p, hsa-miR-675, hsa-miR-920, and downregulated miRNAs: hsa-let-7a, hsa-let-7b*, hsa-let-7c, hsa-let-7d*, hsa-let-7f, hsa-let-7g, hsa-let-7i, hsa-miR-106b*, hsa-miR-126, hsa-miR-1299, hsa-miR-130a, hsa-miR-151-5p, hsa-miR-18a*, hsa-miR-182, hsa-miR-183, hsa-miR-186, hsa-miR-192, hsa-miR-20a, hsa-miR-208a, hsa-miR-22*, hsa-miR-500, hsa-miR-500*, hsa-miR-501-5p, hsa-miR-502-5p, hsa-miR-502-3p, hsa-miR-505*, hsa-miR-532-5p, hsa-miR-574-5p, hsa-miR-574-3p, hsa-miR-576-5p, hsa-miR-625, hsa-miR-629, hsa-miR-652, hsa-miR-7, hsa-miR-886-5p, hsa-miR-92a, hsa-miR-93*, hsa-miR-96	
Wang et al. [74]	Microarray revealed 17 upregulated miRNAs and 103 downregulated miRNAs in MRI(−) acute stroke patients compared with MRI(+) acute stroke patients, 33 upregulated miRNAs and 36 downregulated miRNAs in MRI(+) acute stroke hsa-miR-106b-5P, hsa-miR-4306, hsa-miR-320e hsa-miR-320d	Plasma/0–3 h 23 patients, 3–6 h 37 patients, 6–12 h 31 patients, 12–24 h 45 patients.	136/116	The inclusion criteria consisted of having IS or TIA and having no history of coronary artery disease.	Patients were excluded if they had received intravenous thrombolytic or anticoagulant therapy before the initial blood samples were collected.	LA 60, SA 51, CE 23, UDN 2	Diagnostic value of hsa-miR-106b-5P, hsa-miR-4306, hsa-miR-320e, and hsa-miR-320d	hsa-miR-106b-5p hsa-miR-4306 increased, hsa-miR-320e and hsa-miR-320d decreased which are associated with diagnostic value	NA
Tian et al. [75]	hsa-mir-4454, hsa-mir-140-3p, hsa-mir-106b-5p, hsa-mir-25-3p, hsa-mir-16-5p, hsa-mir-223-3p, hsa-mir-484, hsa-mir-130b-3p, hsa-mir-151a-3p, hsa-mir-130a-3p, hsa-mir-93-5p, hsa-mir-107	Plasma/6 h	40/30	Inclusion criteria: time duration from stroke onset to admission was less than 6 h.	Patients with immune disease, trauma, coronary heart disease, organ failure, tumor, and infection were excluded from the study.	LA 10, SA 8, CE 14, UN 7, ODE 1	Diagnostic and prognostic value of miR-16	MiR-140, miR-106b, miR-130a, miR-16, miR-223, miR-93, miR-484, miR-25, miR-130b, miR-107, and miR-151 were upregulated and miR-4454 was downregulated	Plasma concentrations of miR-16 were related to TOAST criteria, OCSP criteria, and the prognosis of HACI patients
Tiedt et al. [76]	hsa-let-7b-3p, hsa-let-7d-3p, hsa-let-7f-5p, hsa-let-7i-5p, hsa-miR-1, hsa-miR-16-2-3p, hsa-miR-17-3p, hsa-miR-17-5p, hsa-miR-18a-3p, hsa-miR-18a-5p, hsa-miR-20a-5p, hsa-miR-26b-5p, hsa-miR-92a-3p, hsa-miR-99b-5p, hsa-miR-101-3p, hsa-miR-125a-5p, hsa-miR-125b-5p, hsa-miR-126-5p, hsa-miR-130a-3p, hsa-miR-140-3p, hsa-miR-143-3p, hsa-miR-181a-5p, hsa-miR-193a-5p, hsa-miR-378a-3p, hsa-miR-423-3p, hsa-miR-532-5p, hsa-miR-660-5p, hsa-miR-3158-3p, hsa-miR-3158-5p, hsa-miR-3184-5p hsa-miR-3688-3p, hsa-miR-3688-5p	Plasma/24 h, 48 h, 72 h, 90th day.	260/160	IS and TIA patients were enrolled to the study.	Patients with active malignant disease, inflammatory or infectious diseases, surgery within the last three months and prior medication with low-molecular or unfractionated heparin within the last month were excluded. Further, for the discovery sample patients with prior use of antiplatelet medication within the last month, a history of myocardial infarction, stroke, or TIA, or signs for silent CNS infarction on neuroimaging were also excluded. For the replication sample, patients with prior medication with low-molecular or unfractionated heparin within the last month were excluded.	LA 61, SA 18, CE 79, UN 96	Diagnostic value of miR-125a-5p, miR-125b-5p, miR-143-3p	MiR-125a-5p, miR-125b-5p, and miR-143-3p were upregulated.	The transformed infarct volumes of IS patients (N = 188) were correlated with expression levels of miR-125a-5p, miR-125b-5p, and miR-143-3p.
Mick et al. [77]	ex-RNAs by RNASeq (331 miRNAs, 97 piRNAs, and 43 snoRNAs) for RT-qPCR analysis: miR-877-5p, miR-124-3p, miR-320d, snoRNA SNO1402, hsa-miR-656-3p, hsa-miR-941	Plasma/NA	2763 participants included from (Framingham Heart Study; Offspring Cohort Exam 8), unbiased next-generation sequencing conducted using plasma from 40 participants from the cohort.	Subjects were diagnosed with stroke based on review of medical records, including relevant hospitalizations, and clinic reported events by at least 2 neurologists agreeing on one of the following manifestations: definite cerebrovascular accident, atherothrombotic infarction of the brain, cerebral embolism, intracerebral hemorrhage, or subarachnoid hemorrhage.	NA	NA	NA	Observational study	miR-877-5p, miR-124-3p, and miR-320d) and one snoRNA (SNO1402) were independently associated with prevalent stroke, hsa-miR-656-3p and hsa-miR-941 were significantly associated with incident stroke
Chen et al. [64]	Let-7b, miR-23a, miR-126, miR-15a, miR-16, miR-17-5p, miR-19b, miR-29b, miR-339-5p, miR-21, miR-221, miR-32-3p, miR-106-5p, miR-532-5p, miR-145, miR-146b, miR-210	Serum/24 h	128/102	Patients with IS within 24 h after symptoms were enrolled to the study.	Exclusion criteria were being under 18 years old, being on thrombolytic or anticoagulant therapies, intracerebral hemorrhage or hemorrhagic transformation, other complicating neurological or neuropsychological diseases, cancer, comorbidity with proinflammatory conditions, and clinical signs of infection at any time during the study.	NA	Diagnostic value for miR-146b. There is no significance of the expression of serum miRNAs among 5 IS groups in this study. It was suggested that miR-146b may be a biomarker for IS but not for separating subtypes of IS.	MiR-21, miR-145, miR-29b and miR-146b were significantly upregulated and miR-23a and miR-221 levels were significantly downregulated	Positive significant correlation was found between serum miR-146b level and plasma hs-CRP, infarct volume and NIHSS score, and serum IL-6 of patients.
Wu et al. [78]	754 miRNAs were screened, 71 miRNAs were upregulated and 49 miRNAs were downregulated in IS patients	Serum/NA	50/50 then it was confirmed with a larger cohort 177 IS, 81 TIA patients and 42 controls	IS and TIA patients were enrolled to the study. The diagnosis of IS was based on the acute occurrence of focal neurological deficit lasting for more than 24 h and was confirmed by the positive findings of brain CT and MRI.	Patients with a history of hemorrhagic infarction, peripheral arterial occlusive diseases, chronic liver/kidney diseases, primary/metastatic neoplasms or other malignant diseases were excluded.	NA	Prognostic value for miR-23b-3p and miR-29b-3p	MiR-23b-3p, miR-29b-3p, miR-181a-5p and miR-21–5p were markedly increased in IS patients. MiR-23b-3p, miR-29b-3p and miR-181a-5p were also significantly elevated in TIA patients	MiR-23b-3p levels in IS patients were positively related with discharge mRS scores, while miR-23b-3p and miR-29b-3p levels in IS patients were negatively related with discharge BI scores.

Abbreviations: CE, cardioembolic stroke; LA, large artery stroke; SA, small artery stroke; ST, stroke types; TP, time point of blood sampling; UDN, stroke due to undetermined cause, ODE, other determined etiologies; miR, microRNA; hs-CRP, high sensitivity C-reactive protein; HDL, high density lipoprotein; LDL, low density lipoprotein; NIHSS, National Institutes of Health Stroke Scale; IL-6, interleukin 6; TOAST, Trial of Org 10,172 in Acute Stroke Treatment; OCSP, Oxfordshire Community Stroke Project; HACI, hyperacute cerebral infarction; mRS, Modified Rankin Scale; BI, Barthel index; CSF, cerebrospinal fluid; CNS, central nervous system; NA, no data; IS, ischemic stroke; ACI, acute cerebral infraction; MCI, massive cerebral infarction; HS, hemorrhagic stroke; MRI, Magnetic resonance imaging; MRA, magnetic resonance angiography; CT, computed tomography; ApoA1, apolipoprotein A1; MMP-9, matrix metallopeptidase 9; NF-κB, Nuclear factor-κB; TIA, transient ischemic attack; IGF-1, Insulin-like growth factor 1; h, hour; w, week.

**Table 2:** The results of area under the receiver operating characteristic curves from human studies.

Ref	MicroRNA	AUC		Specificity	Sensitivity
Long et al. [41]	miR-30a	24 h	0.91	80%	94%
		1 week	0.91	84%	93%
		4 weeks	0.92	84%	90%
		24 weeks	0.93	84%	92%
	miR-126	24 h	0.92	84%	92%
		1 week	0.94	86%	90%
		4 weeks	0.93	84%	92%
		24 weeks	0.92	82%	92%
	Let-7b	24 h	0.93	84%	92%
		1 week	0.92	84%	90%
		4 weeks	0.92	86%	92%
		24 weeks	0.91	80%	89%
Wu et al. [48]	miR-15a,	0.698		NA	NA
	miR-16,	0.820			
	miR-17-5p	0.784			
	miR-15a + miR-16 + miR-17-5p	0.845			
Peng et al. [43]	Let-7e	0.86		73.4%	82.8%
	miR-338	0.63		53.2%	71.9%
Huang et al. [44]	Let-7e-5p	0.82		NA	NA
Jin F. and Xing J. [53]	miR-126	0.654		NA	NA
	miR-130a	0.642			
	miR-222	0.584			
	miR-218	0.624			
	miR-185	0.601			
	miR-126 + miR-130a + miR-222 + miR-218 + miR-185	0.767			
Chen et al. [61]	miR-223	0.859		78.8%	84%
Sepramaniam et al. [73]		Cohort 1	Cohort 2	NA	NA
	miR-125b-2*	0.95	0.85		
	miR-27a*	0.89	0.86		
	miR-422a	0.92	0.86		
	miR-488	0.87	0.86		
	miR-627	0.84	0.76		
Tian et al. [75]	miR-16 (overall patients)	0.775		87%	69.7%
	miR-16 (LAA patients)	0.952		91.3%	100%
Wang et al. [74]		Total	MRI(+)	NA	NA
	hsa-miR-106b-5p	0.999	0.962		
	hsa-miR-4306	0.877	0.952		
	hsa-miR-320e	0.953	0.981		
	hsa-miR-320d	0.977	0.987		
Jia et al. [55]	CRP	0.794		NA	NA
	CRP+ miR-145	0.896			
	CRP + miR-23a	0.816			
	CRP + miR-221	0.819			
Tsai et al. [57]	miR-21/miR-221 (traditional risk factors)	0.93		NA	NA
Ji et al. [62]	miR-9	0.8026		NA	NA
	miR-124	0.6976			
Tiedt et al. [76]	miR-125a-5p + miR-125b-5p + miR-143-3p	0.663 (IS vs. TIA)			
	miR-125a-5p + miR-125b-5p + miR-143-3p + IL6 + NSE	0.661 (IS vs. TIA)			
Chen et al. [64]	miR146b	0.776		NA	NA
	CRP	0.782			
	IL-6	0.684			
	CRP+miR146b	0.863			
	IL-6+miR146b	0.819			
	CRP+IL-6+miR146b	0.866			

Abbreviations; MiRNA, microRNA; AUC, area under curve; NA, no data; CRP, C-reactive protein; LAA, large artery atherosclerosis; IL-6, interleukin 6; NSE, neuron specific enolase; MRI, magnetic resonance imaging; IS, ischemic stroke; TIA, transient ischemic attack.

## Supplementary Materials

The following are available online at http://www.mdpi.com/2073-4409/7/12/249/s1.
