# Supplementary material for: MicroRNAs as Diagnostic and Prognostic Biomarkers in Ischemic Stroke—A Comprehensive Review and Bioinformatic Analysis
Source: Cells. 2018 Dec 6;7(12):249. doi: 10.3390/cells7120249 (PMC6316722; doi:10.3390/cells7120249)
Supplement: Supplementary file 1 [file cells-07-00249-s001.pdf]

Table S1. The key words used for screening the GO terms

**blood coagulation**	**platelet activation**	**inflammation process**
coagulation	platelet activation	inflammatory response
activation of blood coagulation via clotting cascade	platelet dense granule organization	negative regulation of inflammatory response
blood coagulation	platelet degranulation	positive regulation of inflammatory response
blood coagulation, common pathway	platelet alpha granule lumen	inflammatory response to antigenic stimulus
blood coagulation, extrinsic pathway	platelet-derived growth factor receptor binding	regulation of inflammatory response
blood coagulation, fibrin clot formation	positive regulation of cell proliferation by VEGF-activated platelet derived growth factor receptor signaling pathway	negative regulation of cytokine production involved in inflammatory response
blood coagulation, intrinsic pathway	negative regulation of platelet-derived growth factor receptor-beta signaling pathway	negative regulation of connective tissue replacement involved in inflammatory response wound healing
negative regulation of blood coagulation	negative regulation of platelet activation	negative regulation of inflammatory response to wounding
negative regulation of coagulation	platelet-derived growth factor receptor signaling pathway	acute inflammatory response to antigenic stimulus
positive regulation of blood coagulation	cellular response to platelet-derived growth factor stimulus	positive regulation of cytokine production involved in inflammatory response
positive regulation of coagulation	platelet alpha granule	cytokine production involved in inflammatory response
protein C inhibitor-coagulation factor V complex	negative regulation of platelet aggregation	acute inflammatory response
protein C inhibitor-coagulation factor Xa complex	platelet-derived growth factor binding	positive regulation of acute inflammatory response
protein C inhibitor-coagulation factor XI complex	platelet formation	chronic inflammatory response
regulation of blood coagulation	platelet morphogenesis	negative regulation of inflammatory response to antigenic stimulus
regulation of blood coagulation, intrinsic pathway	platelet dense tubular network membrane	negative regulation of chronic inflammatory response
response to anticoagulant	platelet-derived growth factor receptor-beta signaling pathway	leukocyte chemotaxis involved in inflammatory response
	platelet dense granule lumen	positive regulation of respiratory burst involved in inflammatory response
	platelet alpha granule membrane	regulation of chronic inflammatory response

	regulation of platelet aggregation	connective tissue replacement involved in inflammatory response wound healing
	platelet aggregation	chronic inflammatory response to antigenic stimulus
	platelet activating factor metabolic process	positive regulation of chronic inflammatory response to non-antigenic stimulus
	serotonin secretion by platelet	
	positive regulation of platelet-derived growth factor production	
	platelet dense tubular network	

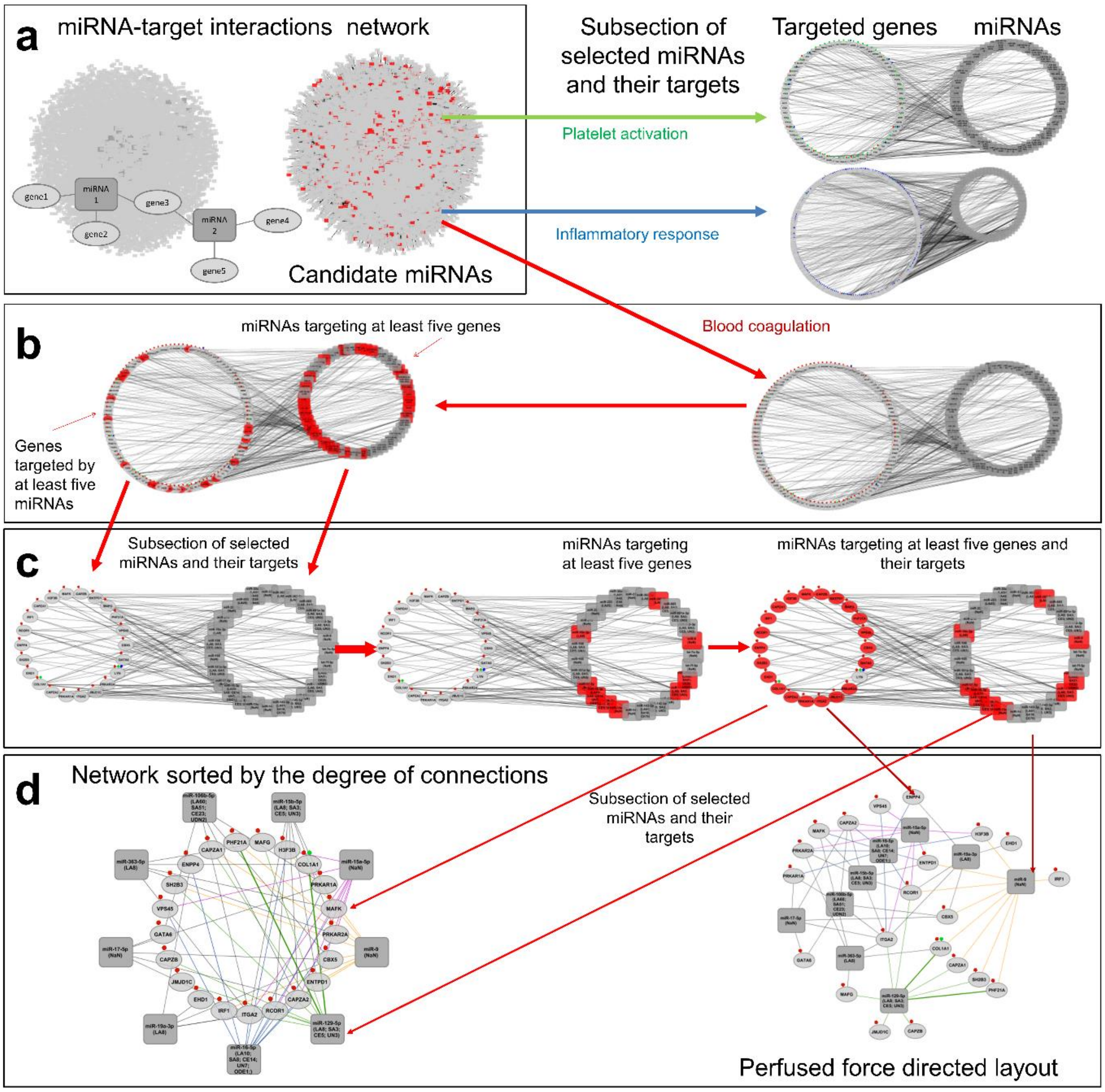

Figure S1. Bioinformatic workflow
